# Effects of Concurrent Training on Resting and Progressive Exercise Metabolism in Breast Cancer Survivors with Metabolic Syndrome Risk Factors

**DOI:** 10.3390/nu18121882

**Published:** 2026-06-11

**Authors:** Cristian Álvarez, Alejandra Soto, Valeska Subiábre, Paulina Ibacache-Saavedra, Luis Peñailillo, Cristóbal Durán-Marín, Igor Cigarroa, Cézane P. Reuter, Anelise R. Gaya, Pedro Delgado-Floody, David C. Andrade, Emmanuel Gomes Ciolac, Mikel Izquierdo, Gabriel Rojas-Rojas

**Affiliations:** 1 Exercise and Rehabilitation Sciences Institute, School of Physical Therapy, Faculty of Rehabilitation Sciences, Universidad Andres Bello, Santiago 7591538, Chile; paulina.ibacache@unab.cl (P.I.-S.); luis.penailillo@unab.cl (L.P.); 2 Tecnología Médica, Facultad de Medicina, Universidad Andres Bello, Concepción 4030000, Chile; asoto@unab.cl (A.S.); valeska.subiabre@unab.cl (V.S.); 3 Doctorate in Rehabilitation Science Program, Exercise and Rehabilitation Sciences Institute, Faculty of Rehabilitation Sciences, Universidad Andres Bello, Santiago 7591538, Chile; c.durnmarn@uandresbello.edu; 4 Escuela de Kinesiología, Facultad de Ciencias de la Salud, Universidad Católica Silva Henríquez, Santiago 8580000, Chile; icigarroac@ucsh.cl; 5 Graduate Program in Health Promotion, University of Santa Cruz do Sul (UNISC), Santa Cruz do Sul 96816-501, Brazil; cezanereuter@unisc.br; 6 School of Physical Education and Physiotherapy, Federal University of Pelotas, Pelotas 96055-630, Brazil; anegaya@gmail.com; 7 Department of Physical Education, Sport and Recreation, Universidad de La Frontera, Temuco 4811230, Chile; pedro.delgado@ufrontera.cl; 8 Exercise Applied Physiology Laboratory, Centro de Investigación en Fisiología y Medicina de Altura (FIMEDALT), Departamento Biomédico, Facultad de Ciencias de la Salud, Universidad de Antofagasta, Antofagasta 1240000, Chile; david.andrade@uantof.cl; 9 Exercise and Chronic Disease Research Laboratory, Department of Physical Education, School of Sciences, Sao Paulo State University (UNESP), Bauru 17033-360, Brazil; emmanuel.ciolac@unesp.br; 10 Navarrabiomed, Hospital Universitario de Navarra (HUN), Universidad Pública de Navarra (UPNA), IdiSNA, 31008 Pamplona, Spain; mikel.izquierdo@gmail.com; 11 CIBER of Frailty and Healthy Aging (CIBERFES), Instituto de Salud Carlos III, 28029 Madrid, Spain

**Keywords:** concurrent training, high-intensity interval training, resistance training, breast cancer, fat oxidation, carbohydrate oxidation, metabolism

## Abstract

**Background/Objectives**: Cancer pathophysiology involves metabolic disturbances, and exercise improves muscle metabolism, but little is known about the effect of concurrent high-intensity interval plus resistance training (CT_HIIT+RT_) on the resting and exercise metabolism of breast cancer survivors with risk factors related to metabolic syndrome (MetS). The purpose of this study was to determine the effects of 8 weeks of CT_HIIT+RT_ on the fat and carbohydrate (CHO) metabolism during rest and progressive exercise post-exercise intervention in breast cancer survivors with histories of high- and low-volume chemotherapy. **Methods**: An experimental study was conducted in which (*n* = 21) women breast cancer survivors (age: 58.7 ± 8.7 years) were divided by history of high- (HV_chemo_, ≥8 to 16 sessions, *n* = 10) or low-volume (LV_chemo_, ≤7 chemo sessions, *n* = 11) chemotherapy. The fat (RFAT_ox_) and CHO oxidation during 10 min of resting was measured (i.e., including the area under curve [AUC] points within this time), as well as the fat (ExFAT_ox_) and CHO (ExCHO_ox_) oxidation during progressive exercise, before and after 8 weeks of CT_HIIT+RT_ by indirect calorimetry. Additionally, risk factors related to MetS were described from pre- to post-intervention. **Results**: At rest, the HV_chemo_ group showed significant increases in the AUC of RFAT_ox_ (∆ + 23.7, *p* < 0.0001), similar to the LV_chemo_ group (∆ + 14.0, *p* < 0.0001). The HV_chemo_ group showed significant increases in the AUC of RCHO_ox_ (∆ + 109.7, *p* < 0.0001), similar to the LV_chemo_ group (∆ + 107.5, *p* < 0.0001). During progressive exercise, the ExFAT_ox_ was increased in the HV_chemo_ group (∆ + 0.12 g/min, *p* = 0.006), similar to the ExCHO_ox_ (∆ + 0.52 g/min, *p* = 0.013) of this group. The proportion of subjects categorized with MetS were significantly reduced in blood pressure, Glu and triglyceride components. **Conclusions**: Eight weeks of CT_HIIT+RT_ may promote favorable metabolic and cardiometabolic adaptations in breast cancer survivors regardless of the chemotherapy exposure volume. However, these findings should be interpreted cautiously due to the small sample size and lack of a true non-exercise control group.

## 1. Introduction

Cancer is the second leading cause of death worldwide [1]. Breast cancer alone accounts for approximately 12% of cancer-related mortalities [2]. Multiple factors increase cancer risk, including genetics, physical inactivity, unhealthy diet, obesity, and socioeconomic disadvantages [3]. Physical inactivity alone contributes to 4.1% of cancer diagnoses among 15 cancer types in the United States [4]. In Chile (Latin America), breast cancer increased in parallel with mortality from 2022 (rate 13.6%; 1049 deaths) to 2025 (16.9%; 1705 deaths) [5].

Following breast cancer diagnosis and subsequent surgical intervention, chemotherapy (chemo) remains an essential treatment strategy during acute survivorship [6]. However, even if chemo is effective, it affects normal cells, leading to metabolic syndrome (MetS) [3,7]. For example, 18 months of chemo has been shown to elevate fasting plasma glucose (Glu) by approximately (+20 mg/dL), systolic blood pressure by (+6 mmHg), diastolic blood pressure by (+7 mmHg), waist circumference by (+4 cm), and total cholesterol by (+16.4 mg/dL), thereby promoting a worse decline in metabolic health [8]. Specific chemo agents, particularly Anthracyclines and Trastuzumab (Herceptin), further increase cardiotoxicity [i.e., symptoms related to increases in arterial stiffness and blood pressure] and body fat and decrease skeletal muscle mass (i.e., sarcopenia) [9]. Sarcopenia is frequently reported among breast cancer patients, particularly by those receiving cyclin-dependent kinase 4 and 6 inhibitors [10], highlighting the need for targeted exercise training modalities to enhance skeletal muscle mass (SMM). Maintaining SMM significantly mitigates chemo-induced metabolic disturbances and improves overall metabolic health [11]. Notably, under insulin-stimulated conditions, SMM accounted for approximately 80% of the carbohydrate (CHO) uptake following a 75 g glucose (Glu) load [12,13]. Thus, another secondary consequence of chemo-induced side effects includes metabolic dysfunction, which alters substrate utilization (i.e., fat and CHO utilization) under resting or exercise conditions [14]. In fact, changes in substrate utilization (i.e., using less fat and more CHO) measured by indirect calorimetry have been shown to be more strongly associated with MetS [15]. Other cancer-related consequences of chemo treatment in breast cancer survivors include fatigue, anxiety, pain, lymphedema, depressive symptoms, compromised bone health, and diminished physical condition [16].

Exercise training is critical for maintaining SMM and controlling fat and CHO utilization and is recognized as a physical therapy approach [17], being recommended by the American College of Sports Medicine (ACSM) [16] and the American Diabetes Association (ADA) [18] for improving cardiometabolic health. In brief, high-intensity interval training (HIIT) is an exercise modality of wide capacity used to improve Glu control in diabetes subjects and improve oxidative metabolism [19,20], and it has been shown to increase cardiorespiratory fitness and alleviate fatigue and emotional symptoms in breast cancer patients [21]. Resistance training (RT) for 12 weeks is also effective at reducing Glu levels under diabetes diagnosis [22]. Bettariga et al. [23] reported that after 12 weeks of RT or HIIT exercise in breast cancer survivors, RT increased muscle strength in the chest press exercise (+4.7 kg), and HIIT increased cardiorespiratory fitness (∆ + 2.8 mL/kg/min), with both modalities promoting significant body fat decreases. In contrast, six months of concurrent training using moderate-intensity continuous plus RT (CT_MICT+RT_) increased cardiorespiratory fitness in these cohorts [24]. However, there is a scarcity of knowledge about the effects of concurrent training with a low volume of HIIT plus RT (CT_HIIT+RT_) on the resting and progressive exercise post-exercise intervention on the substrate metabolism in breast cancer survivors. The purpose of this study was to determine the effects of 8 weeks of CT_HIIT+RT_ on the fat and carbohydrate (CHO) metabolism during rest and progressive exercise post-exercise intervention in breast cancer survivors with histories of high- and low-volume chemotherapy. We hypothesized that 8 weeks of CT_HIIT+RT_ would improve the fat utilization during resting (RFAT_ox_) and progressive (ExFAT_ox_) exercise conditions after the intervention period. Moreover, regardless of the resting CHO utilization behavior, we expected that CHO utilization would increase during progressive exercise (regardless of which group the patient was assigned to (higher or lower chemotherapy exposure).

## 2. Materials and Methods

### 2.1. Patients

This study was part of the Onco–Vascular Exer Study, an experimental clinical trial conducted with 21 adult women breast cancer survivors. Participants were recruited from different cancer social groups associated with the university. Subjects of interest underwent preliminary screening, including detailed sociodemographic and clinical evaluations. According to their chemo history, participants were allocated to either the high-volume-chemo (HV_chemo_) or low-volume-chemo (LV_chemo_) group. All participants underwent metabolic and other clinical assessments at baseline and after completing eight weeks of CT_HIIT+RT_, performed three times per week. This study adhered to the Declaration of Helsinki, and all participants provided written informed consent. This study was registered at ClinicalTrials.gov (NCT06766903) and was approved by the Bioethical Committee of the Universidad Andres Bello (Approval Acta 005/2024) on 12 April 2024.

The inclusion criteria were as follows: (i) breast cancer diagnosis; (ii) history of having received at least one session of chemo treatment; (iii) a body mass index [BMI] between 18.6 and 39.9 kg/m^2^; (iv) age between 30 and 75 years (functionally independent); (v) the possibility of maintaining around 8 weeks of commitment to adherence to exercise and clinical interventions at the university lab; and (vi) controlled prediabetes or diabetes, prehypertension hypertension, metabolic syndrome, fatty liver disease or hypercholesterolemia [25]. Exclusion criteria included: (i) history of abnormal electrocardiogram; (ii) uncontrolled cancer hormonal therapy; (iii) uncontrolled pharmacotherapy for emergencies (^SOS^) to manage pain, such as morphine patches, morphine droplets; (iv) diagnosis or history of cardiovascular disease (e.g., other than controlled hypertension); (v) vasculopathy; (vi) history of uncontrolled stage 3 hypertension or hypertensive crisis; (vii) diabetes-related complications, such as varicose ulcers, nephropathies; (viii) SMM abnormalities (e.g., knee or hip arthrosis, non-cancer-related muscle pain); (ix) use of pharmacotherapy influencing weight loss; (x) being active or under exercise training programs or involved in exercise in the past three months; (xi) respiratory disease (e.g., chronic obstructive disease); (xii) kidney disease; (xiii) condition of pregnancy; (xiv) smoking behavior or dependence on other substances.

The number of participants required for this experimental study, which compared two groups, was estimated from the expected change in body fat %. Thus, to determine how many participants were needed, we focused on the expected change in body fat %. Earlier studies using similar interventions have reported a standard deviation of about (±3.4%) for this outcome, and we used this value as the basis for our calculation. With this SD data and setting the usual thresholds of a 0.05 alpha level and 80% statistical power, the estimation suggested that at least (*n* = 8) participants in each HV_chemo_ and LV_chemo_ group would be enough to detect a meaningful difference in how body fat % changed over time. The final sample size comprised (*n* = 10) participants in the HV_chemo_ group and (*n* = 11) in the LV_chemo_ group. The CONSORT flow chart is described in (Supplementary Material Figure S1; https://figshare.com/s/97dd7e2dc7fa9aadd30d).

### 2.2. Categorization of Chemotherapy History Volume

Considering all subjects screened, those who reported being treated with 8 chemo sessions were categorized as HV_chemo_, and those who declared between 1 and 7 chemo sessions were categorized as LV_chemo_, similar to previous studies [26]. Thus, the group distribution was developed intentionally to balance the groups according to those in the HV_chemo_ group and those in the LV_chemo_ group.

### 2.3. Anthropometric and Body Composition Outcomes

Weight was measured using BIA equipment (InBody^TM^ 270, Seoul, Republic of Korea). Height (cm) was measured using a stadiometer (Health o Meter^TM^ Professional, Sunbeam Products, Inc., Chicago, IL, USA). Waist circumference was measured using an inextensible tape (SECA^TM^, Chino, CA, USA). The BMI was calculated using both weight plus height [27]. The delta changes (∆) in weight, BMI, and waist circumference were calculated from the pre- to post-test time. All measurements were performed in the laboratory in the morning. The general characteristics of the patients are shown in (Supplementary Material Figure S2; https://figshare.com/s/522df8acf39d5259f7d4).

### 2.4. Resting and Exercising Metabolism by Indirect Calorimetry

Subjects were asked to consume a normal breakfast for each evaluation, and they were also asked to wear comfortable clothes and refrain from drinking caffeinated and alcoholic beverages and from performing intense physical exercise over the preceding 24 h. Thus, the participants arrived in the morning at the lab and were instructed to rest in a supine position on a stretcher for 20 min. For clarity purposes, the resting substrate oxidation variables were identified as the RFAT_ox_ (i.e., fat utilization) and RCHO_ox_ (i.e., CHO utilization), whereas the exercise substrate oxidation variables were identified as the ExFATox and ExCHO_ox_ (i.e., fat and CHO substrate utilization during progressive exercise). Thus, during the 10 min, the RFAT_ox_ and RCHO_ox_ were assessed using indirect calorimetry by breath-by-breath oxygen volume (VO_2_) and carbon dioxide (CO_2_) gas exchange measurements (Ultima Series^TM^, Cardiorespiratory Diagnostic Systems, CardiO_2_, MGC, Saint Paul, MN, USA). The data were analyzed using Breeze Suite^TM^ v. 8.6 SP7 cardiorespiratory diagnostic software. Additionally, the areas under the curve for fat oxidation (AUC RFAT_ox_) and carbohydrate oxidation (AUC RCHO_ox_) were calculated using each minute-by-minute data point obtained during the resting period before and after the intervention.

Following the resting assessment, the participants performed a modified Astrand progressive cycling exercise test on an electronically braked cycle ergometer (Ergoselect 200, ERGOLINE^TM^, Bitz, Germany), as previously used [28,29]. Before testing, the participants received safety instructions and were screened for adequate CHO intake, hydration status, and recent physical activity. Individuals identified as physically inactive or reporting no moderate-to-vigorous physical activity in the previous week were provided with a familiarization session without exercise testing, and the exercise test was conducted during a subsequent visit. During the Astrand test, heart rate was continuously monitored using a wrist-worn device (Polar A370, Kempele, Finland) [30]. The exercise load was increased progressively by 25 Watt every two minutes until volitional fatigue. Peak heart rate (HR_peak_) and power output (PO) in watts were monitored at each stage of the test. Subsequently, in the data stored, the maximal fat (ExFAT_ox_) and carbohydrate (ExCHO_ox_) oxidation during the exercise test were recorded [31,32], as well as the power output at each maximal fat (PO_MFATox_) and CHO (PO_MCHOox_) oxidation before and after 8 weeks of CT_HIIT+RT_ intervention.

### 2.5. Resting Metabolism by Capillary Glucose and Total Cholesterol

The participants arrived in the morning after having a standardized breakfast for their pre- and post-measurements. Capillary (Cap) blood samples were collected from the fingertips to assess the Cap glucose (_Cap_Glu), Cap total cholesterol (_Cap_Tc), and Cap triglyceride (_Cap_Tg) concentrations using a calibrated portable analyzer (Accutrend^TM^ Plus COBAS, Roche Diagnostics, Mannheim, Germany).

### 2.6. Presence or Absence of Risk Factors Related to Metabolic Syndrome

Using standardized criteria for categorization, we included four out of five MetS risk factors: elevated waist circumference (WC, ≥88 cm), systolic blood pressure (SBP, ≥130 mmHg), and diastolic blood pressure (DBP, ≥90 mmHg), which do not exhibit substantial variability under fasting or postprandial conditions, in addition to triglycerides (Tg, ≥150 mg/dL) and glucose (Glu, ≥100 mg/dL). These latter two risk factors were measured after the ingestion of each participant’s habitual breakfast (i.e., under postprandial-like conditions) and using a Cap sampling mode [33]. After the intervention, these four MetS risk factors were checked and described as proportions and percentages (%) of “Risk Factor Present” or “Risk Factor Absent” in participants per each group and before and after the intervention.

### 2.7. Concurrent Training Program

All participants were offered the opportunity to participate in the 8-week CT_HIIT+RT_ program performed three times per week. The first two weeks consisted of familiarization with the minimal exercise volume and gradual progression. Participants’ feedback on their pain and tolerance was also collected. In the HIIT stage, stationary bicycles (Impulse^TM^, model PS 300, Qingdao, China) were used. The protocols included five intervals of 60 s at 80–100% HR_peak_ during weeks 1–4 and seven intervals during weeks 5–8, with inter-interval recovery at ≤70% HR_peak_. Heart rate was monitored with Polar^TM^ A370 devices, and the HR_peak_ was adjusted session by session, and thus the % of exercise was maintained in the HIIT intervals by this adjustment. The RT included five (weeks 1–5) to seven (weeks 6 and 7) 60 s sets of strength exercises: bicep curls (2 sets), shoulder presses (2 sets), and back exercises (1 set) in weeks 1–5, and additional step (1 set) and flexion/extension of gastrocnemius (1 set) exercises in weeks 6 and 7, performed at 20–50% of the estimated 1-repetition maximum (1 RM) at pre-test, and regulated session by session. Rest periods were individualized based on the modified Borg Rating of Perceived Exertion (target: 1–3 out of 10). The total session duration ranged from 30 to 40 min. The exercise prescriptions adhered to the ACSM volume-per-week guidelines [34]. The study protocol can be seen in (Supplementary Material Figure S3; https://figshare.com/s/b2f628c9568fddfd7991).

### 2.8. Statistical Analysis

All data are reported as means ± standard deviation (SD). Normality and homoscedasticity assumptions were assessed using the Shapiro–Wilk test. A two-way ANOVA with a repeated-measures (Group x Time) test was applied to indicate inter- and intra-group differences in the data under Gaussian distribution. Additionally, Sidak’s *post hoc* test was performed to identify significant differences by the ANOVA analyses. Effect sizes were calculated using Cohen’s *d* and interpreted as follows: <0.20 (negligible); 0.20–0.49 (small); 0.50–0.79 (moderate); and ≥0.80 (large) [35]. The area under the curve (AUC) was calculated to describe both the AUC RFAT_ox_ and AUC RCHO_ox_ using point-by-point data during 1 to 10 min of resting in each group. To describe the categorical data of “Risk Factor Present” or “Risk Factor Absent” related to MetS and compare the proportions from the pre- vs. post-test, the Chi square test was applied. Delta changes (∆) from the post-minus pre-test were calculated to describe some characteristics of the sample. Statistical analyses were performed using Prism 8.0 software (Graph Pad, San Diego, CA, USA), with significance set at *p* ≤ 0.05. The statistics for the presence/absence of MetS risk factors were developed using SPSS^TM^ software 29 version for Windows (IBM SPSS Inc., Chicago, IL, USA).

## 3. Results

### 3.1. Anthropometric and Body Composition Changes

No significant differences were observed between the HV_chemo_ and LV_chemo_ groups at baseline. Waist circumference significantly decreased in both groups: HV_chemo_ (from 102.8 ± 13.4 cm to 98.3 ± 11.4 cm, *p* = 0.015, *d* 0.54) and LV_chemo_ (from 97.1 ± 11.5 cm to 90.5 ± 12.6 cm, *p* = 0.001, *d* 0.70) (Table 1).

### 3.2. Fat and CHO Oxidation at Rest and During Progressive Exercise

**Figure 1:**
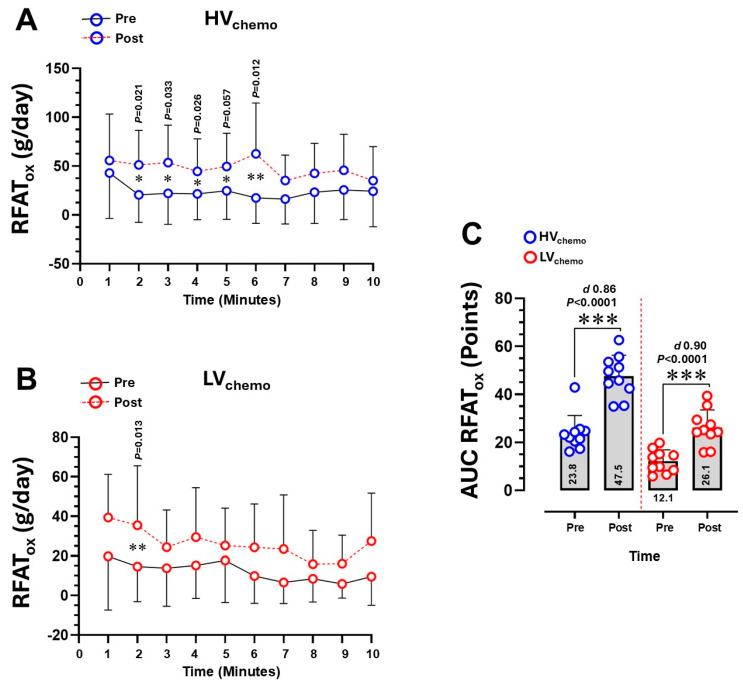
Fat oxidation during a resting period before and after 8 weeks of concurrent training in female breast cancer survivors with histories of high-volume chemotherapy (**A**) and low-volume chemotherapy (**B**). Panel (**C**) illustrates the area under the curve (AUC) of fat oxidation over the 10 min resting period. Groups are defined as: (HV_chemo_) high-volume-chemotherapy group; (LV_chemo_) low-volume-chemotherapy group. Outcomes are described as: (RFAT_ox_) resting fat oxidation; (AUC FAT_ox_) area under the curve of fat oxidation during ten minutes of resting. (*) Significant differences between pre- and post-tests at *p* < 0.05. (**) Significant differences between pre- and post-tests at *p* < 0.01. (***) Denotes significant differences between pre- and post-tests at *p* < 0.0001. (*d*) Denotes Cohen’s *d* effect size at *p* < 0.05. Values expressed in (g/day) can be converted to g/min by dividing the reported value by 1440 (g/min).

During resting, significant increases in the RFAT_ox_ were observed in the HV_chemo_ group at minute 2 (*p* = 0.021; Δ + 30.8 g/day [+150.2%]); minute 3 (*p* = 0.033; Δ + 31.5 g/day [+143.1%]); minute 5 (*p* = 0.057; Δ + 24.8 g/day [+100%]); and minute 6 (*p* = 0.012; Δ + 45.2 g/day [+259.7%]) (Figure 1A). In the LV_chemo_ group, significant increases in the RFAT_ox_ were observed only at minute 2 (*p* = 0.013; Δ + 20.8 g/day [+142.4%]) (Figure 1B). The area under the curve (AUC) analysis for the RFAT_ox_ during the 10 min resting period showed significant increases in both the HV_chemo_ (23.8 ± 9.1 to 47.4 ± 9.3 g/day; *p* < 0.0001; *d* 0.86) and LV_chemo_ (12.1 ± 11.0 to 26.1 ± 19.0 g/day; *p* < 0.0001; *d* 0.90) groups (Figure 1C).

**Figure 2:**
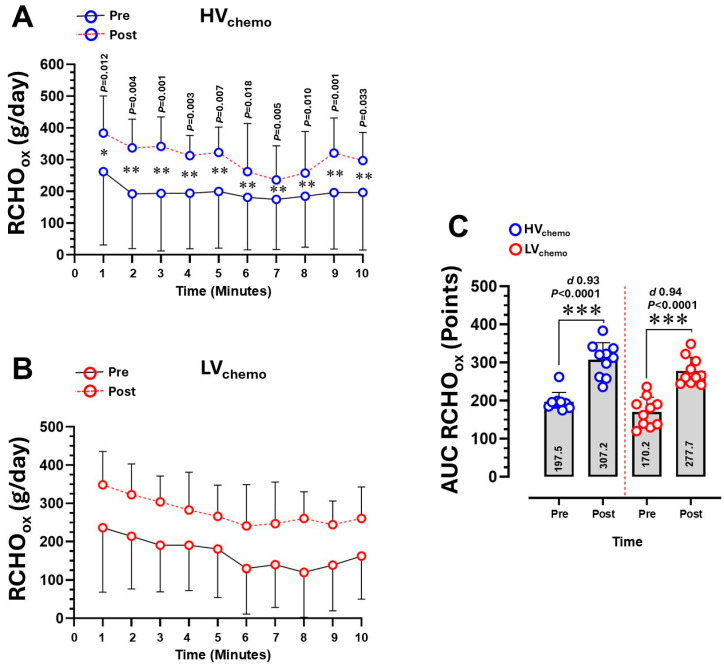
Carbohydrate oxidation during rest before and after 8 weeks of concurrent training in female breast cancer survivors with histories of high-volume (**A**) and low-volume (**B**) chemotherapy. Panel (**C**) shows the area under the curve for carbohydrate oxidation during ten minutes of rest. Groups are described as follows: (HV_chemo_) high-volume-chemotherapy group; (LV_chemo_) low-volume-chemotherapy group. Outcomes are defined as: (RCHO_ox_) resting carbohydrate oxidation; (AUC CHO_ox_) area under the curve of carbohydrate oxidation during ten minutes of rest. (*) Denotes significant differences between pre- and post-tests at *p* < 0.05. (**) Denotes significant differences between pre- and post-tests at *p* < 0.01. (***) Denotes significant differences between pre- and post-tests at *p* < 0.0001. (*d*) Denotes Cohen’s *d* effect size at *p* < 0.05. Values expressed in (g/day) can be converted to g/min by dividing the reported value by 1440 (g/min).

In the HV_chemo_ group, there were significant RCHO_ox_ increases during the 10 min periods: minute 1 (*p* = 0.012; Δ + 121.1 [+45.1%]); minute 2 (*p* = 0.004; Δ + 144.9 [+75.3%]); minute 3 (*p* < 0.001; Δ + 148.2 [+76.5%]); minute 4 (*p* = 0.003; Δ + 118.8 [+61.2%]); minute 5 (*p* = 0.007; Δ + 123.2 [+61.7%]); minute 6 (*p* = 0.018; Δ + 81.1 [+44.8%]); minute 7 (*p* = 0.005; Δ + 61.2 [+35.0%]); minute 8 (*p* = 0.010; Δ + 73.0 [+39.4%]); minute 9 (*p* = 0.001; Δ + 124.5 [+63.4%]); and minute 10 (*p* = 0.033; Δ + 100.4 [+50.9%]) (Figure 2A). In the LV_chemo_ group, there were no changes in the RCHO_ox_ (Figure 2B). AUC analysis for the RCHO_ox_ showed significant increases in both groups: HV_chemo_ (197.5 ± 33.2 to 307.2 ± 41.3 g/day; *p* < 0.0001; *d* 0.93) and LV_chemo_ (170.2 ± 35.0 to 277.7 ± 26.0 g/day; *p* < 0.0001; *d* 0.94) (Figure 2C).

(Supplementary Material Figure S3) shows the RFAT_ox_ and RCHO_ox_ across the 10 min resting period, together with their respective AUC RFATox and AUC RCHO_ox_ values, combining participants from both groups (HV_chemo_ and LV_chemo_) into a single analysis. Despite observing an increase in the number of significant comparisons for the RCHO_ox_ and a reduction in significant comparisons for the RFAT_ox_ at specific time points, the supplementary figure confirms that there was a significant increase from pre- to post-intervention in both the AUC RFAT_ox_ and AUC RCHO_ox_ when considering the total sample of (*n* = 21) subjects (see Supplementary Material Figure S3; https://doi.org/10.6084/m9.figshare.32311722).

**Figure 3:**
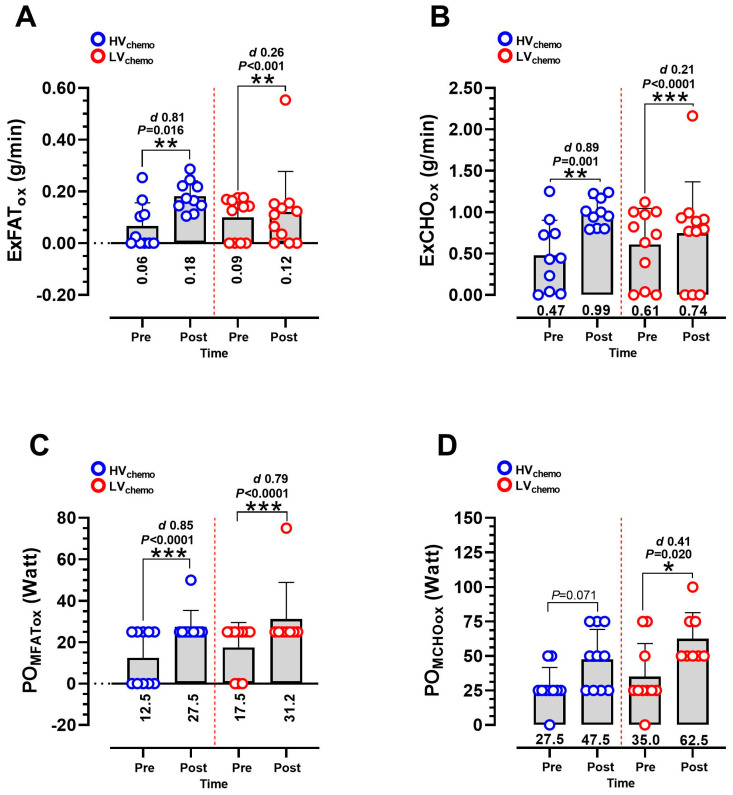
Fat (**A**) and CHO (**B**) oxidation during exercise and power output at which maximal FAT (**C**) and CHO oxidation (**D**) occurred before and after eight weeks of concurrent training in female breast cancer survivors with histories of high- and low-dose-chemotherapy treatment. Groups are described as (HV_chemo_) high-dose-chemotherapy group and (LV_chemo_) low-dose-chemotherapy group. Outcomes are described as: (ExFAT_ox_) fat oxidation during exercise, (ExCHO_ox_) carbohydrate oxidation during exercise, (PO_MFATox_) power output at maximal fat oxidation during exercise, and (PO_MCHOox_) power output at maximal carbohydrate oxidation during exercise. (*) Denotes significant differences between pre- and post-tests at *p* < 0.05. (**) Denotes significant differences between pre- and post-tests at *p* < 0.01. (***) Denotes significant differences between pre- and post-tests at *p* < 0.0001. (*d*) Denotes Cohen’s *d* effect size. All bold values denote significant statistical changes/differences at *p* ≤ 0.05.

During the exercise test, significant increases in the ExFAT_ox_ were observed in the HV_chemo_ group (0.06 ± 0.01 to 0.18 ± 0.01 g/min, *p* = 0.016, d 0.81) and LV_chemo_ group (0.09 ± 0.01 to 0.12 ± 0.01 g/min, *p* < 0.001, *d* 0.26) (Figure 3A). Similarly, significant increases in the ExCHO_ox_ were observed in the HV_chemo_ group (0.47 ± 0.42 to 0.99 ± 0.17 g/min; *p* = 0.001, *d* 0.89) and LV_chemo_ group (0.61 ± 0.46 to 0.74 ± 0.34 g/min, *p* < 0.0001, *d* 0.21) (Figure 3B). The power output corresponding to the PO_MFATox_ significantly increased in the HV_chemo_ group from pre- to post-intervention (12.5 ± 13.1 to 27.5 ± 7.9 Watts; *p* < 0.0001, *d* 0.85), as well as in the LV_chemo_ group (17.5 ± 14.0 to 27.5 ± 17.8 Watts; *p* < 0.0001, *d* 0.79) (Figure 3C). The PO_MCHOox_ was increased significantly in the LV_chemo_ group (35.0 ± 24.1 to 62.5 ± 18.9 Watts; *p* = 0.020, *d* 0.41) but not in the HV_chemo_ group (Figure 3D).

(Supplementary Material Figure S4) shows the ExRFAT_ox_ and RCHO_ox_ across the 10 min resting period, together with their respective AUC RFATox and AUC RCHO_ox_ values, combining participants from both groups (HV_chemo_ and LV_chemo_) into a single analysis. Despite observing an increase in the number of significant comparisons for the RCHO_ox_ and a reduction in significant comparisons for the RFAT_ox_ at specific time points, the supplementary figure confirms that there was a significant increase from pre- to post-intervention in both the AUC RFAT_ox_ and AUC RCHO_ox_ when considering the total sample of (*n* = 21) subjects (see Supplementary Material Figure S4; https://figshare.com/s/21f96b0ae3e63629f43e).

### 3.3. Capillary Glucose, Cholesterol and Triglycerides

**Figure 4:**
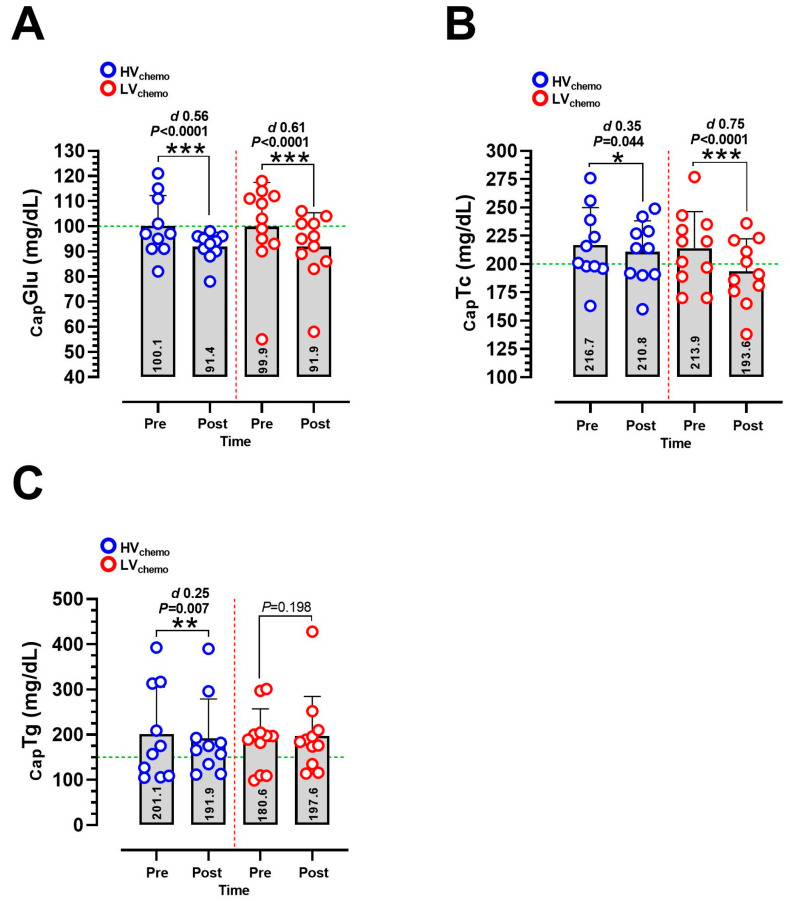
Capillary glucose (**A**), total cholesterol (**B**) and capillary measured triglycerides (**C**) levels before and after 8 weeks of concurrent training in female breast cancer survivors with histories of high- and low-dose-chemotherapy treatment. Groups are described as: (HV_chemo_) high-dose-chemotherapy group; (LV_chemo_) low-dose-chemotherapy group. Outcomes are described as: (Cap) capillary, (Glu) glucose, (Tc) total cholesterol, and (Tg) triglycerides. (---) The green line denotes the cutoff point for each fasting glucose level: (>100 mg/dL hyperglycemia in panel A) and (>200 mg/dL high cholesterol in panel B). (*) Denotes significant differences between pre- and post-tests at *p* < 0.05. (**) Denotes significant differences between pre- and post-tests at *p* < 0.01. (***) Denotes significant differences between pre- and post-tests at *p* < 0.0001. (*d*) Denotes Cohen’s *d* effect size.

Significant changes were detected comparing the before and after measurements of the _Cap_Glu of the HV_chemo_ group (100.1 ± 12.0 to 91.4 ± 5.3 mg/dL, *p* < 0.0001, ∆ − 8.7 mg/dL, *d* 0.56) to those of the LV_chemo_ group (99.9 ± 14.0 to 91.9 ± 12.1 mg/dL, *p* < 0.0001, ∆ − 8.0 mg/dL, *d* 0.61) (Figure 4A). In terms of the _Cap_Tc, there were significant reductions in both the HV_chemo_ (216.7 ± 27.0 to 210.8 ± 19.2 mg/dL, *p* < 0.0001, ∆ − 5.9 mg/dL, *d* 0.35) and LV_chemo_ (213.9 ± 26.0 to 193.6 ± 24.4 mg/dL, *p* < 0.0001, ∆ − 20.1 mg/dL, *d* 0.75) groups (Figure 4B). In terms of the _Cap_Tg levels, there were observed significant reductions in the HV_chemo_ group (201.1 ± 102.1 to 191.9 ± 88.5 mg/dL, *p* = 0.007, ∆ − 9.2 mg/dL, *d* 0.25) but not in the LV_chemo_ group (Figure 4C).

### 3.4. Presence or Absence of Metabolic Syndrome-Related Risk Factors

There were significant differences in three risk factors screened before and after the CT_HIIT+RT_ intervention (Table 2). In terms of the increased blood pressure, in the HV_chemo_ group, there were significant changes in the proportion of “Risk Factor Absent” from pre- to post-test (30.0 to 80.0%, *p* = 0.042), similar to the LV_chemo_ group (45.4 to 81.8%, *p* = 0.047) (Table 2). Similarly, in terms of the increased glucose, measured by the _Cap_Glu outcome, in the HV_chemo_ group, the proportion of “Risk Factor Absent” from pre- to post-test changed (70.0 to 100.0%, *p* < 0.0001), similar to the LV_chemo_ group (45.4 to 63.6%, *p* < 0.0001) (Table 2). The _Cap_Tg group showed similar changes in the proportion of “Risk Factor Absent” from pre-to post-test (20.0 to 40.0%, *p* < 0.0001); however, the LV_chemo_ group remained unchanged (Table 2).

## 4. Discussion

This study aimed to determine the effects of 8 weeks of CT_HIIT+RT_ on the fat and carbohydrate (CHO) metabolism during the rest and post-exercise period in breast cancer survivors with histories of high- and low-volume chemo. The primary findings indicate the following: (i) CT_HIIT+RT_ may have enhanced metabolic flexibility in breast cancer survivors after the intervention, where both the HV_chemo_ and LV_chemo_ groups showed higher resting FAT and CHO utilization, as reflected by the AUC RFAT_ox_ and AUC RCHO_ox_ responses (Figure 1 and Figure 2). (ii) In contrast, during the progressive volitional exercise test, increases in both fat and CHO utilization, assessed through ExFAT_ox_ and ExCHO_ox_ outcomes, were observed only in breast cancer survivors from the HV_chemo_ group (Figure 3A,B), and these findings may indicate a differential metabolic responsiveness to CT_HIIT+RT_ according to prior chemo exposure; however, given the study design and sample size, these results should be considered with caution and require confirmation in larger controlled trials. (iii) After the 8 weeks of CT_HIIT+RT_ intervention, the proportion of subjects categorized as “Risk Factor Absent” was significantly increased in blood pressure, glucose and Tg in the HV_chemo_ group (Table 2) and LV_chemo_ group (despite Tg), suggesting a potential reduction in MetS risk factors in this sample of female cancer survivors, independent of their history of high- or low-chemo-volume exposure.

Under breast cancer diagnosis, chemo is the first-line therapeutic tool in the reduction of tumor cells, but at the same time, it has known adverse metabolic effects, such as increased fasting glucose, blood pressure, and cholesterol levels, that increase the MetS risk in adult cancer survivors [8]. Exercise training interventions have been recommended by relevant institutions, such as the ACSM, to counteract these adverse effects, which include metabolic disturbances [16]. Regarding our first results of increased AUC RFAT_ox_ and AUC RCHO_ox_ utilization during resting in both the HV_chemo_ and LV_chemo_ groups (Figure 1 and Figure 2), our results demonstrate that CT_HIIT+RT_ may be associated with favorable changes in the resting substrate utilization in breast cancer survivors within 10 min of resting. These findings suggest that independent of a higher- or lower-chemo-volume history, the CT_HIIT+RT_ program may reflect adaptive metabolic responses in resting by increasing fat and CHO oxidation, suggesting potential increases in resting substrates. Importantly, the favorable changes in the resting substrate utilization observed in both the HV_chemo_ and LV_chemo_ groups were further supported by the pooled-sample analysis, which can be found in (Supplementary Material Figure S3; https://doi.org/10.6084/m9.figshare.32311722), that demonstrates significant increases in both the AUC RFAT_ox_ and AUC RCHO_ox_ from pre- to post-intervention when participants from both groups were combined (*n* = 21). Roudi et al. [15] reported, in a cross-sectional study (*n* = 1.015) where men (57.6%) and women (39.4%) participated, that the resting fat oxidation in the women group was similar between those participants with “Risk Factor Present” in comparison with those with “Risk Factor Absent”, observing, therefore, a phenotype predominantly inclined toward a more glycolytic metabolism, which is largely associated with a more favorable environment for the emergence of abnormal cells such as cancer.

Regarding our second result, in which we found significant improvements in both fat and CHO oxidation during exercise (ExFAT_ox_ and ExCHO_ox_) in both the HV_chemo_ and LV_chemo_ groups (Figure 3A,B), it has been described that some chemo, particularly cyclin-dependent kinase 4 and 6 inhibitors, exacerbates sarcopenia and impairs the ability of SMM to oxidize fats efficiently [10]. Along this line, it is widely known that SMM preservation is crucial, as approximately 80% of glucose uptake under insulin-stimulated conditions occurs in the skeletal muscle as glycogen is stored [12]. From this, we speculate that exercise training may contribute to mitochondrial adaptations, reversing chemotherapy-induced declines in oxidative capacity, similar to acute and short-term exercise training studies reporting molecular changes in adults without cancer or in those with type 2 diabetes [20,36,37]. Nevertheless, these mechanistic interpretations remain speculative because molecular markers and control comparisons were not evaluated in the present study. Our data indicate that CT_HIIT+RT_ can positively affect CHO utilization at rest and during progressive exercise, promoting an optimized metabolic state, especially in breast cancer survivors with a history of increased chemo volume. The observed increase in CHO utilization during exercise is further supported by the pooled-sample analysis, when participants from both groups were analyzed together (*n* = 21), and significant increases were observed in both the ExFAT_ox_ and ExCHO_ox_ following the intervention, reinforcing the evidence for favorable exercise-induced adaptations in substrate utilization (see Supplementary Material Figure S4; https://figshare.com/s/21f96b0ae3e63629f43e). A recent literature study review from Puurand et al. [38] has reported that exercise training induces tumor suppression and modulates cancer energy metabolism by releasing extracellular vesicles, transferring microRNA to the circulation, proteins and metabolites, thereby altering glycolysis and oxidative phosphorylation and promoting more metabolic plasticity.

Additional exercise benefits from the CT_HIIT+RT_ program were the increases in the PO_MFATox_ and PO_MCHOox_, as almost all groups (i.e., despite the HV_Chemo_ group in this last outcome) showed increases in the PO performance during cycling. Bauer et al. [39], after 12 weeks of MICT or HIIT, showed that the PO by cycling was increased by (+17 Watt) and (+27 Watt), respectively, for each exercise mode. From our results, we observed that after 8 weeks of CT_HIIT+RT_, the PO_MFATox_ increased by (∆ + 15 W) in the LV_chemo_ group, and the PO_MCHOox_ increased by (∆ + 20 W) in the HV_chemo_ group and by (∆ + 27.5 W) in the LV_chemo_ group, which agrees with the previous literature, but less volume intervention was favored in our current study. From a functional perspective, the CT_HIIT+RT_ program through HIIT intervals may suggest potential functional improvements in lower-limb power performance, enhancing performance in the activities of daily living. The increases in power output (watts) observed in both the PO_MFATox_ and PO_MCHOox_ were also corroborated by the pooled-sample analysis. When participants from both groups were combined, similar significant improvements were observed, further supporting the robustness of these exercise-induced adaptations (see Supplementary Material Figure S4; https://figshare.com/s/21f96b0ae3e63629f43e).

Regarding our third result—the increase in subjects categorized as “Risk Factor Absent” independent of their history of chemo volume treatment (Table 2)—Thomas et al. [40] reported that after 6 months of MICT in an exercise group (i.e., three supervised and two unsupervised exercise sessions per week at 60–80% of the predicted heart rate) and a control group, the exercise group decreased the Glu (∆ − 1.3 mg/dL) in comparison with the control group, which reported an increase in this outcome (∆ + 0.6 mg/dL), but as is widely known in dose–response, when the authors reported the data by those who were better “adherers” to exercise, there were major reductions in the Glu (∆ − 1.4 mg/dL), waist circumference (∆ − 2.4 cm), and Tg (∆ − 3.1 mg/dL). Our results, although derived from lower-volume and shorter-duration exercise, were superior in waist circumference reduction (∆~5 cm reduction), and despite not showing statistical significance, were more clinically relevant for decreasing _Cap_Glu (∆~9.1 mg/dL reduction) and _Cap_Tg (∆~13.1 mg/dL reduction), considering both the HV_chemo_ and LV_chemo_ groups. It is of relevance to mention that both groups were hyperglycemic or at the limit of this condition, hypercholesterolemic, and under hypertriglyceridemia, all outcomes of the metabolic control in which CT_HIIT+RT_ could play a role (Figure 4). Finally, the reduction in _Cap_Tc observed in both groups highlights the need for early cardiometabolic screening and lifestyle interventions, especially in breast cancer survivors under extended hormonal therapy, given that fatty liver disease is a frequent comorbidity associated with longer therapy duration. These adaptations underscore the importance of targeted supervised exercise programs for breast cancer survivors to stimulate both skeletal muscle mass and the cardiorespiratory system for more effective substrate oxidation and thereby decrease the risk factors associated with MetS diagnosis.

## 5. Limitations and Strengths

This study has several limitations: (i) We did not specify the type of chemo treatment applied, nor the volume of chemo sessions per group. (ii) Resting substrate metabolic and _Cap_Glu, _Cap_Tc, and _Cap_Tg measurements were not carried out in fasting conditions, and blood samples were developed by Cap samples. (iii) We did not separate groups or categories by menopausal development. (iv) Our study lacked a true control group. (v) The exercise test was progressive/volitional and not under steady-state conditions. (vi) Although we reported the RFAT_ox_ and RCHO_ox_ in units of (g/day) because our metabolic equipment is factory-configured to display these variables in this format, both FAT and CHO variables can also be calculated in g·min^−1^, as is indicated in the footnote of each figure. Specifically, conversion from (g/day) to (g·min^−1^) should be performed by dividing the (g/day) by 1440. (vii) Also, it is important to report that although participants from both groups attended the RFAT_ox_ and RCHO_ox_ assessment sessions as well as the progressive exercise evaluation (pre–post) developed under “habitual” breakfast conditions to obtain ExFAT_ox_ and ExCHO_ox_ outcomes, we did not require the consumption of a standardized breakfast; therefore, we declare that dietary intake was not controlled during the intervention protocol. Instead, we suggested maintaining the regular intake of macronutrients on the day prior to and the day of evaluation, with special emphasis on CHO intake, since it is well established that muscle and liver glycogen are fundamental metabolic substrates for vigorous-intensity exercise, such as progressive exercise performed until voluntary exhaustion and the CT_HIIT+RT_ intervention applied in this study. Accordingly, we used the ASA24 instrument (ASA24 Dietary Assessment Tool) and observed no significant between-group changes from pre- to post-intervention in the macronutrient intake in the HV_chemo_ group (Kcal, pre: 1543.0 ± 442.2 vs. post: 1324.0 ± 213.5; Protein, pre: 22.5 ± 8.9 vs. post: 18.2 ± 10.1; CHO, pre: 40.7 ± 13.3 vs. post: 47.7 ± 8.6; FAT, pre: 36.5 ± 6.1 vs. post: 33.5 ± 2.3) or LV_chemo_ group (Kcal, pre: 1645.9 ± 651.9 vs. post: 1345.0 ± 289.7; Protein, pre: 22.7 ± 9.5 vs. post: 22.5 ± 5.6; CHO, pre: 43.5 ± 14.5 vs. post: 52.4 ± 7.7; FAT, pre: 33.7 ± 9.0 vs. post: 25.0 ± 7.3). Therefore, this instrument supports the interpretation that the observed changes in the FAT or CHO utilization are not attributable to dietary changes. (viii) This study used a pre-/post-intervention design without a non-exercise control group, limiting the ability to establish causal relationships between the intervention and the observed outcomes.

This study also had notable strengths: (i) adherence to the CT_HIIT+RT_ exercise intervention among the participating breast cancer survivors was good, highlighting the feasibility of implementing short-term, concurrent training programs in this clinical population; (ii) the structured supervised exercise protocol allowed for accurate familiarization and session-by-session monitoring; and (iii) the substrate quantification was applied during resting and exercise conditions, which allowed for a wider evaluation of the energy used by this poorly studied sample.

## 6. Conclusions

Eight weeks of CT_HIIT+RT_, combining abbreviated HIIT with low-load RT, may promote favorable metabolic adaptations in breast cancer survivors with previous histories of high- and low-volume-chemo exposure. Both groups demonstrated higher resting FAT and CHO utilization following the intervention, whereas increases in substrate utilization during progressive volitional exercise appeared to occur primarily in the HV_chemo_ group, suggesting the possible chemo role. Additionally, a greater proportion of participants were classified as “Risk Factor Absent” for high blood pressure, glucose and triglycerides, part of the MetS outcomes. Collectively, these findings suggest that CT_HIIT+RT_ may contribute to improvements in metabolic flexibility and cardiometabolic health in breast cancer survivors. However, given that our study lacked a true non-exercise control group and included a relatively small sample size, these findings should be interpreted with caution, and future controlled studies are needed to confirm these observations.

## Figures and Tables

**Table 1:** Anthropometric characteristics of participants.

Outcomes	Time	HV_chemo_	Within-Group *p* Value	LV_chemo_	Within-Group *p* Value	Between-Group *p* Value, Cohen’s *d*
Anthropometric						
Age (y)	Pre	58.1 ± 10.4		59.3 ± 7.0		*p* = 0.980
Height (m)	Pre	1.56 ± 0.03		1.56 ± 0.03		*p* = 0.766
Weight (kg)	Pre	70.8 ± 12.5		66.9 ± 11.2		*p* = 0.608, 0.03
	Post	71.7 ± 12.4		66.9 ± 11.3		
	∆	0.9	*p* = 0.131, 0.23	0.0	*p* = 0.970, 0.00	
Body mass index (kg/m^2^)	Pre	28.8 ± 4.5		27.3 ± 5.3		*p* = 0.642, 0.02
	Post	29.2 ± 4.4		27.3 ± 5.2		
	∆	0.4	*p* = 0.124, 0.24	0.0	*p* = 0.999, 0.00	
Waist circumference (cm)	Pre	102.8 ± 13.4		97.1 ± 11.5		*p* = 0.577, 0.04
	Post	98.3 ± 11.4 *		90.5 ± 12.6 **		
	∆	−4.5	*p* = 0.015, 0.54	−6.6	*p* = 0.001, 0.70	
Body fat (%)	Pre	43.2 ± 6.3		39.8 ± 7.8		*p* = 0.283, 0.06
	Post	44.7 ± 5.1		41.1 ± 7.6		
	∆	1.5	*p* = 0.585	1.3	*p* = 0.676	

Data are shown as means ± SD. Groups are described as (HV_chemo_) high-volume chemotherapy and (LV_chemo_) low-volume chemotherapy. (∆) Denotes delta changes in absolute data from post-minus pre-test. (*) Denotes significant within-group differences from pre-test at *p* ≤ 0.05. (**) Denotes significant within-group differences from pre-test at *p* ≤ 0.001.

**Table 2:** Changes in risk factors related to metabolic syndrome after 8 weeks of concurrent high-intensity interval plus resistance training in women breast cancer survivors.

Outcomes	Time	Risk Factor Present/Absent	HV_chemo_	*d* Somers	LV_Chemo_	*d* Somers
(*n* = )			10		11	
MetS Criteria						
Increased waist circumference (cm)	Pre	Risk Factor Present, *n* = (%)	9 (90.0)	*p* = 0.236	10 (90.9)	*p* = 0.247
		Risk Factor Absent, *n* = (%)	1 (10.0)		1 (9.0)	
	Post	Risk Factor Present, *n* = (%)	9 (90.0)		10 (90.9)	
		Risk Factor Absent, *n* = (%)	1 (10.0)		1 (9.0)	
Increased high blood pressure (mmHg)	Pre	Risk Factor Present, *n* = (%)	7 (70.0)	***p* = 0.042**	6 (54.5)	***p* = 0.047**
		Risk Factor Absent, *n* = (%)	3 (30.0)		5 (45.4)	
	Post	Risk Factor Present, *n* = (%)	2 (20.0)		2 (18.1)	
		Risk Factor Absent, *n* = (%)	8 (80.0)		9 (81.8)	
Increased glucose (mg/dL)	Pre	Risk Factor Present, *n* = (%)	3 (30.0)	***p* < 0.0001**	6 (54.5)	***p* < 0.0001**
		Risk Factor Absent, *n* = (%)	7 (70.0)		5 (45.4)	
	Post	Risk Factor Present, *n* = (%)	0 (0)		4 (36.3)	
		Risk Factor Absent, *n* = (%)	10 (100)		7 (63.6)	
Increased triglycerides (mg/dL)	Pre	Risk Factor Present, *n* = (%)	8 (80.0)	***p* < 0.0001**	7 (63.6)	***p* < 0.0001**
		Risk Factor Absent, *n* = (%)	2 (20.0)		4 (36.3)	
	Post	Risk Factor Present, *n* = (%)	6 (60.0)		9 (81.8)	
		Risk Factor Absent, *n* = (%)	4 (40.0)		2 (18.1)	

Data are shown as means ± SD. Groups are described as (HV_chemo_) high-dose chemotherapy and (LV_chemo_) low-dose chemotherapy. Categories are described as: (Risk Factor Present) risk factor present and (Risk Factor Absent) risk factor absent. Bold values denote significant differences into “Risk Factor Present” and “Risk Factor Absent” categories comparing pre- vs. post-test within-group differences at *p* ≤ 0.05.

## Data Availability

The data are freely available in the repository at the following URL; https://figshare.com/s/c1238709f5fff8444a74 (accessed on 5 June 2026).

## Supplementary Materials

The following supporting information can be downloaded at: https://figshare.com/s/97dd7e2dc7fa9aadd30d, https://figshare.com/s/522df8acf39d5259f7d4, https://figshare.com/s/b2f628c9568fddfd7991, https://figshare.com/s/21f96b0ae3e63629f43e (accessed on 5 June 2026), Figure S1: CONSORT flow chart study design. Figure S2: General characteristics of the participants before the Intervention. Figure S3: Resting fat and CHO oxidation in breast cancer survivors before and after 8 weeks of concurrent training. Figure S4: Exercise fat oxidation, exercise CHO oxidation, peak power output at maximal fat utilization, and peak power output at maximal CHO utilization during progresive/incremental exercise in adults breast cancer survivors after 8 weeks of concurrent training.

## Abbreviations

The following abbreviations are used in this manuscript:

ACSM	American College of Sports Medicine
ADA	American Diabetes Association
AUC	Area Under the Curve
BIA	Bioelectrical Impedance Analysis
BMI	Body Mass Index
Cap	Capillary
_Cap_Glu	Capillary Glucose
_Cap_Tc	Capillary Total Cholesterol
_Cap_Tg	Capillary Triglycerides
CHO	Carbohydrate
CONSORT	Consolidated Standards of Reporting Trials
CO_2_	Carbon Dioxide
CT_HIIT+RT_	Concurrent High-Intensity Interval Training + Resistance Training
CT_MICT+RT_	Concurrent Moderate-Intensity Continuous Training + Resistance Training
DBP	Diastolic Blood Pressure
ExCHO_ox_	Exercise Carbohydrate Oxidation
ExFAT_ox_	Exercise Fat Oxidation
Glu	Glucose
HIIT	High-Intensity Interval Training
HR_peak_	Peak Heart Rate
HV_chemo_	High-Volume Chemotherapy
LV_chemo_	Low-Volume Chemotherapy
MetS	Metabolic Syndrome
MICT	Moderate-Intensity Continuous Training
PO	Power Output
POMCHO_ox_	Power Output at Maximal Carbohydrate Oxidation
POMFAT_ox_	Power Output at Maximal Fat Oxidation
RFAT_ox_	Resting Fat Oxidation
RCHO_ox_	Resting Carbohydrate Oxidation
RPE	Rating of Perceived Exertion
RT	Resistance Training
SBP	Systolic Blood Pressure
SD	Standard Deviation
SMM	Skeletal Muscle Mass
Tg	Triglycerides
VO_2_	Oxygen Consumption
W	Watts
WC	Waist Circumference
∆	Delta (Change)
